# Factors influencing performance by contracted non-state providers implementing a basic package of health services in Afghanistan

**DOI:** 10.1186/s12939-018-0847-4

**Published:** 2018-10-05

**Authors:** Ahmad Shah Salehi, Abdul Tawab Kawa Saljuqi, Nadia Akseer, Krishna Rao, Kathryn Coe

**Affiliations:** 1 London School of Hygiene and Tropical Medicine, London, UK; 2 The University of Arizona, Tucson, AZ USA; 3 Centre for Global Child Health, The Hospital for Sick Children Toronto and the University of Toronto, Ontario, ON Canada; 4 Bloomberg School of Public Health, Johns Hopkins University, Baltimore, MD USA; 5 Fairbanks School of Public Health, Indiana University- Purdue University Indianapolis, Indianapolis, USA

**Keywords:** Contracting out, Non-state providers, Afghanistan

## Abstract

**Background:**

In 2002 Afghanistan’s Ministry of Public Health (MoPH) and its development partners initiated a new paradigm for the health sector by electing to Contract-Out (CO) the Basic Package of Health Services (BPHS) to non-state providers (NSPs). This model is generally regarded as successful, but literature is scarce that examines the motivations underlying implementation and factors influencing program success. This paper uses relevant theories and qualitative data to describe how and why contracting out delivery of primary health care services to NSPs has been effective.

The main aim of this study was to assess the contextual, institutional, and contractual factors that influenced the performance of NSPs delivering the BPHS in Afghanistan.

**Methods:**

The qualitative study design involved individual in-depth interviews and focus group discussions conducted in six provinces of Afghanistan, as well as a desk review. The framework for assessing key factors of the contracting mechanism proposed by Liu et al. was utilized in the design, data collection and data analysis.

**Results:**

While some contextual factors facilitated the CO (e.g. MoPH leadership, NSP innovation and community participation), harsh geography, political interference and insecurity in some provinces had negative effects. Contractual factors, such as effective input and output management, guided health service delivery. Institutional factors were important; management capacity of contracted NSPs affects their ability to deliver outcomes. Effective human resources and pharmaceutical management were notable elements that contributed to the successful delivery of the BPHS. The contextual, contractual and institutional factors interacted with each other.

**Conclusion:**

Three sets of factors influenced the implementation of the BPHS: contextual, contractual and institutional. The MoPH should consider all of these factors when contracting out the BPHS and other functions to NSPs. Other fragile states and countries emerging from a period of conflict could learn from Afghanistan’s example in contracting out primary health care services, keeping in mind that generic or universal contracting policies might not work in all geographical areas within a country or between countries.

**Electronic supplementary material:**

The online version of this article (10.1186/s12939-018-0847-4) contains supplementary material, which is available to authorized users.

## Background

Afghanistan has experienced profound difficulties over the past decades, especially since the 1978 invasion by the former Soviet Union which led to political instability, pervasive conflict and, at times, outright war. In 1992, the Mujahedeen (groups of religiously-driven warriors) took power, initiating a new period of civil war and inter-Mujahedeen conflicts. From 1996 until November 2001, the Taliban emerged as the ruling group in the country with a limited interest in the development of health systems [1].

In December 2001, a new democratic government was established in Afghanistan with international support. The new government inherited extreme disorder in the health sector. No policies were in place to guide the delivery of services and there was a notable lack of coordination among the many actors working on health. The health sector was characterized by the absence of infrastructure, lack of capacity in the public sector, the shortage of health human resources, and inconsistency in the quality of services being delivered [2]. Health outcomes were poor as a result of the disarray: the maternal mortality rate in Afghanistan at that time was one of the highest in the world (1600/100,000 live births) and the under-five mortality rate was one of the worst in the region (257/1000 live births) [2]. Given these challenges, the development of a functioning health care system, which included a program that prioritized maternal and child health, was deemed by the new government to be critically important.

Six months after the new government took power, in May 2002, the Ministry of Public Health (MoPH) established a Basic Package of Health Services (BPHS) with technical support from donors and international organizations. The BPHS was designed to ensure equitable access to a core set of health services in remote and underserved populations. In recognition of the extent of its problems, the Afghan health sector adopted a new paradigm for operations. While health care services were regarded previously as a state responsibility, in 2002 the MoPH and its development partners decided to contract-out (CO) delivery of vital health care services to non-state providers (NSPs) [3]. This paradigm shift was critically important given that, after decades of war, the newly-established government did not have sufficient capacity to deliver health care to the most underserved in the population.

To rapidly scale up country-wide delivery of the BPHS, the MoPH needed NSPs [4]. NSPs (both formal and informal) already provided a wide range of health care services and had extensive geographic reach. Formal NSPs such as non-governmental organizations (NGOs) had extensive local networks, roots and experience providing health services in districts not controlled by the central government. NGOs—most of which had headquarters in Peshawar, Pakistan—had trained and supported Afghan health providers in many provinces and had gained the trust of communities. These NGOs were well-placed to assume more responsibility for delivering health care services [5].

The MoPH launched BPHS implementation in 2003 with financial support from the United States Agency for International Development (USAID), the World Bank (WB), the European Union (EU) and others in the international community. 31 of 34 provinces were contracted with NSPs and were supported by different donors. As a result, different contracting mechanisms were established to implement the standardized and unified BPHS across the country. The MoPH served as the steward and owner of the program.

More than a decade later, the program’s impact was evident in increased health services coverage (defined in terms of having a health facility within walking distance), from 9% in 2002 to 67% in 2015. The country has also made impressive improvements in health systems performance indicators including maternal and child health [6–8].

Proponents of contracting out in Afghanistan have regarded it as effective in rapidly scaling up health services throughout the country [5, 9–13] but critics have expressed concerns about sustainability and cost effectiveness [14–17]. The factors that have promoted the success of contracting out to NSPs in Afghanistan are not yet well understood. Identifying these factors would provide important lessons for Afghanistan and, more generally, for comparative studies of health systems in fragile states.

Contracting of health services to NSPs is an increasingly prevalent trend in developing countries [18]. Loevinsohn and Harding conducted a comprehensive review of 10 CO mechanisms in low-resource settings. They found that the systems for contracting out needed to be adjusted to address specific needs in each country’s unique context [19, 20]. Moreover, the authors argue, optimal service delivery outcomes are likely to result under the following conditions: when the NSP maintains autonomy from the state; when a focus is placed on outcomes, outputs and cost-effectiveness; and when rigorous evaluation of the contracted-out projects is planned for and conducted on a regular basis.

A few studies have been conducted in Afghanistan on contracting of NSPs. One review discussed contractual factors, such as how partners are selected and what payment mechanisms are used [20]. Though this review focused on the level of quality of care provided by NSPs, and identified some factors associated with variations in quality, it did not explore contextual or institutional factors related to the contracting structure. The present study aims to address this gap in the literature on health system development in Afghanistan with an in-depth evaluation of the factors underlying the successes and continuing challenges facing a health system in transition from post-conflict development to long-term sustainability. The main aim of this study was to identify the contextual, institutional, and contractual factors that influenced CO of NSPs and their performance during the period 2003 to 2013.

## Methods

### Conceptual framework

Our evaluation of Afghanistan’s CO mechanism for BPHS used a conceptual framework developed by Liu et al. as a foundation and a guide for designing the study, developing data collection tools, and analyzing data [21]. Using the Liu et al. framework provided guidance on methodology. Further, it enables comparisons of the situation in Afghanistan with that of other contracting schemes in other contexts that have also been assessed using the same framework. While the specifics of the geographical and historical situation in Afghanistan are unique, adopting a tested and proven framework contributes to the validity of the findings and makes the findings comparable with other situations.

**Fig. 1:**
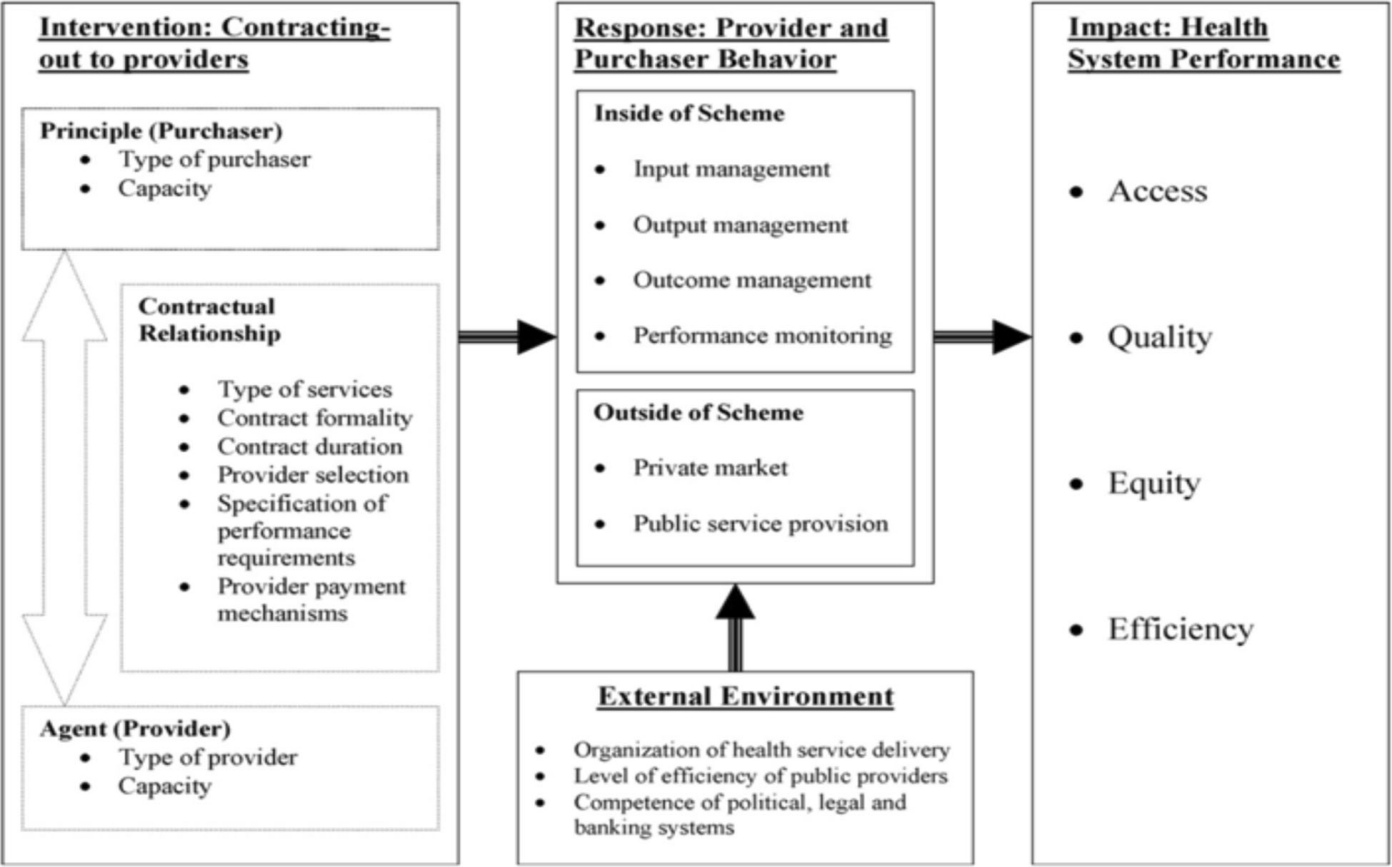
Study Conceptual Framework (Source: Liu et al)

As the Liu et al. framework suggests, this study sought to develop an overview of the contextual, institutional and contractual arrangements that have influenced NSP performance (see Fig. 1) [19, 21]. The study identifies various factors. The study also reviews program performance measures, including “contractual factors”, “contextual factors” (or the external environment) and “institutional factors” (such as hiring and retention of staff, and interactions between providers and purchaser). It sought to capture both intended and unintended effects.

As suggested by Liu et al., the research team conducting this evaluation was not directly involved in the delivery of services in the provinces where the research was carried out. The research team was external to the provinces of interest, and comprised the primary investigator (PI), three co-investigators (COI), six field investigators (FI) and one research coordinator.

In order to represent the varying contexts in Afghanistan, the research was conducted in six provinces: Balkh, Bamyan, Herat, Kabul, Kandahar, and Nangarhar. Aspects of the context included the level of security, geographical features, ethnic variations (i.e. including both Pashtun and Tajik majority provinces), the donors involved (i.e. The World Bank, USAID and EU), and implementing NSP organization.

### Data sources

Three main data collection methods were used: desk review, individual interviews and focus group discussions.

### Desk review

Our literature review explored a range of documents pertaining to the research objectives, including addressing critical issues and major policy arguments related to the role of NSPs in Afghanistan. The desk review incorporated academic papers, gray literature, reports, and official policy documents.

### Qualitative data collection methods: KI interviews and FGDs

Liu et al. note that qualitative data provides rich insights on factors influencing program effectiveness. In line with this comment, this study involved two qualitative data collection methods: in-depth interviews with key national and provincial stakeholders and focus group discussions with local-level stakeholders. We used a purposeful sampling technique to ensure diversity among our respondents [22]. The sampling plan was stratified according to different categories of stakeholders: representatives of the MoPH at both the central and provincial levels, donors, UN agencies, NGOs, health care workers, and health professional associations. The variety allowed the team to explore perceptions and ideas from a diverse group, identifying similarities and divergences across the respondent categories.

The stakeholder and focus group interview guides were developed by the core research team (PI and COIs). The guides were translated into both the Dari and Pashto languages and then cross-translated, piloted, and corrected in order to finalize the study instrument. All interviews and discussions were conducted in either the Dari or Pashto language based on participant preference. Transcriptions were generally made on the same day (or as soon as possible) by the field investigators (FIs), who used both their field notes and recordings to ensure accurate transcription of interviews.

The FIs carried out 36 in-depth interviews and 6 FGDs across all categories of study participants. By design, we focused on health workers’ experiences with the contracting out mechanism; no patients or beneficiaries were included in the study. Table 1 lists all types of interviewees and their affiliations. The interviewees for in-depth interviews were selected using purposeful sampling that considered institutional affiliation (i.e. government or NSP), geographical distribution (representing all the provinces where the study was conducted), and function in the system (policy maker, manager or field level worker). Interviews were conducted at the respondents’ workplaces or other locations where the participants felt comfortable.

**Table 1:** Smpling frame for in depth ke informent interviews (KIs)

Institution	Person(s) to be Interviewed	Number (n)	Reason for Selection
Central Ministry of Public Health (MoPH)	Deputy Minister of Policy and Planning	1	One of four people at MoPH who initiated the contract-out mechanism and continues to oversee the provision of health services by NSPs
	General Director, Policy and Planning	1	Has essential information on contextual, contractual and institutional standards and variations
	Head of Health Management Information System (HMIS)	1	HMIS manages self-reported data from the NSPs on a monthly basis; the department has been involved since the start of the BPHS
	Head of Monitoring & Evaluation (M&E)	1	The department works with the third-party evaluator to develop and oversee the BSC
	Head of Grant and Contract Management Unit (GCMU)	1	GCMU was created specifically for the purpose of facilitating the contracting process; manages procurement, contract management and compliance evaluation of the NSPs for implementation of BPHS
	Provincial Liaison Director	1	Responsible for coordinating provincial-level activities; can provide detail on provinces
Provincial MoPH	Provincial Health Directors	6 (one per province, six provinces)	Provide key information about the context, type of contract and institutional factors for the respective provinces
Third Party Evaluator (Johns Hopkins University and Indian Institute for Health Management and Research)	Evaluator	1	Assessed the performance of BPHS across the country from 2004 to 2013, applying BSC and conducting household surveys
Donors (USAID, WB, EU)	Health team leaders	3 (3 main donors)	Represent the interests and opinions of the three main donors supporting the CO program
Non-state providers (NSPs)	NSP Managers, Kabul (national and international NGOs)	6 (one per province, six provinces)	Understand the type of contract in their province; provide key information about contractual arrangements, context and institutional factors
	Provincial NSP managers	6 (one per province, six provinces)	Province-specific input to contextualize information and get field-level knowledge about each contracted NSP
	Heads of health facilities	12 (two per province, six provinces)	Views of frontline health workers on CO and the contractual, institutional and contextual variations

The participants for the FGDs were also selected through a purposeful sampling process that sought to keep the composition of the FGDs constant across provinces. The members of each FGD were recruited based on predefined criteria and in collaboration with local health authorities. Characteristics of FGD participants are summarized in Table 2. The FGDs were conducted in neutral settings where the participants could freely express themselves.

**Table 2:** Sampling frame for focus droup discussions

Institution	Participant	Number (n)	Reason for Selection
MoPH	Preventive Health Care (PHC) Officer	1	Is aware of all the contractual and service delivery programs in the province
	HMIS Officer	1	Responsible for collection of data from all health facilities at the provincial level and relaying it to central HMIS in Kabul; collects all indicators of BPHS on a monthly basis
	Reproductive Health Officer	1	Provides technical perspective on components of BPHS related to maternal and child health services
	Expanded Program of Immunization (EPI) Officer	1	EPI is the largest health program in the country; officers are experienced and familiar with NSP service provision
NSPs	Deputy Project Manager	1	Oversees monitoring and evaluation of all programs under contract
	Finance Manager	1	Manages inputs and financial mechanisms of NSPs; understands provider payment mechanisms
	Community Supervisor	1	Provides views from community and frontline health workers

The FIs who collected the data were recruited and trained in March and April 2016 by the PI and COIs. The field work, led by the PI and coordinated by the research coordinator, was conducted in June and August 2016. Only the research team had access to the data collected and all interviews and FGDs were assigned codes to preserve anonymity when citing quotations.

### Data analysis

Interview transcriptions and field and diary notes were included in the data analysis. We used content analysis to consider the key issues, elements and outcomes [23]. Topics and concepts were identified, highlighted and placed in categories of association. Themes and statements were coded according to the conceptual framework. Representative quotes were selected and allocated to the relevant classifications. Common viewpoints were described, and particularly important responses were elucidated. Finally, each category was studied and discussed by the research team to develop interpretations of the data that addressed the aims and objectives of the study.

Findings from the interviews and FGDs were triangulated with other data sources in four ways. First, the research team assessed the consistency of the findings generated using the different data collection methods. Second, we examined the consistency of different data from the same method. For instance, we compared multiple sources’ perspectives about the procurement of medical supplies, a topic we discussed with donors, MoPH policy makers and NSPs. Third, multiple analysts reviewed all findings. Fourth, we used various perspectives and theoretical frameworks when interpreting the data. In all cases we made sure that the personal opinions of the research team members were not reported as part of the results.

Combining multiple observers, theories, methods, and data sources helped to avoid problems created by collecting data using only a single method or from only one source. The breadth of perspectives included in the analysis allowed us to comprehensively assess the program and isolate the impact of CO. This was frequently difficult, given the prevalence and severity of problems such as those posed by the environment in parts of Afghanistan [21].

## Results

The results of the study are presented in line with the study’s main objective: to understand the key contextual, contractual and institutional factors that have influenced contracted NSPs’ performance in delivering the BPHS in Afghanistan. These factors are presented in brief in Table 3. Each factor is discussed in detail in the following sections.

**Table 3:** Factors Assessed in this Study

Category of Factor		
Contextual	Contractual	Institutional
• Sociocultural environment	• Contractor selection	*External:* • Performance monitoring
• Political environment	• Contract duration	
• Legal and policy environment	• Contractual requirements	
• Geography	• Types, formality and duration of services to be provided	*Internal:* • Inputs, outputs and outcomes
	• Payment mechanism	

**Fig. 2:**
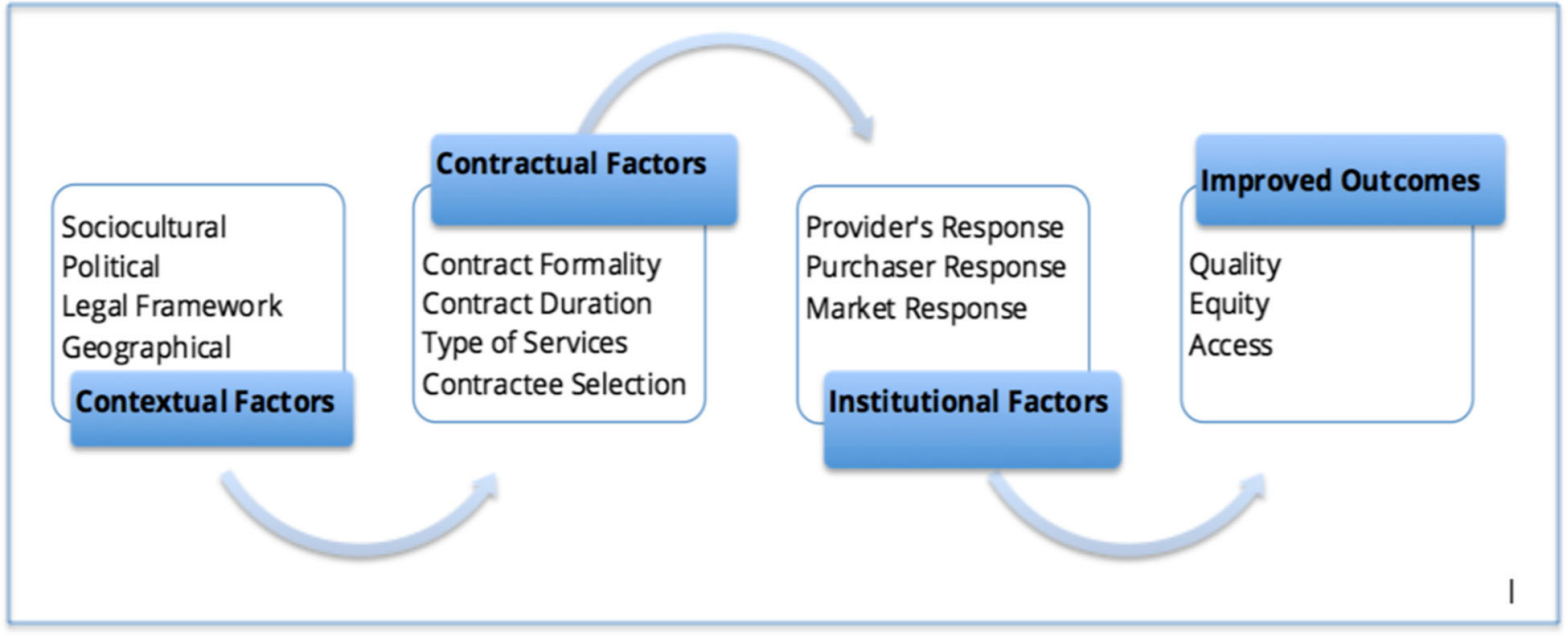
The interaction of contextual, contractual and institutional factors and their relationship with the outcomes

The Liu et al. framework proposes that creating an impact on the health status of a population through contracting out depends on the interplay among three types of factors: contractual, contextual and institutional [21]. When these three sets of factors interact effectively, the health system produces better outcomes, namely: quality, access and coverage of health services. These, in turn, combine to produce the final goals: improved and equitable health status of the population. For example, favorable contextual factors pave the way for a better contractual mechanism to function, which in turn smooths potential pitfalls faced by the institutions involved. The interactions among the three types of factors are therefore as centrally important as identifying and categorizing the factors. These interactions are depicted in Fig. 2.

In the following sections we describe how each factor contributes to improving the performance of the CO mechanism for NSPs contracting-out. We then discuss how their interaction produces impact.

### Contextual factors

Contextual factors include any conditions that create either a conducive or unfavorable environment for an effective program of contracting-out. In Afghanistan, the sociocultural and geographic factors were long-standing conditions. On the other hand, the political changes that followed the fall of the Taliban created a new legal and policy foundation for contracting out. Table 4 summarizes the contextual factors that emerged from the study data.

**Table 4:** Summary of contextual factor findings

Contextual factor	Features (positive (+) or negative (−) impact) affecting contracting-out
Sociocultural environment	• Ethnic and religious traditions and cultures (+/-) • Traditional gender constructs (−) • Social capital and culture of community participation (+)
Political, policy and legal environment	• Capacity and structure of provincial health departments (+/-) • Influence of political leadership on hiring of staff and implementation of services (−) • Conflict and insecurity (−) • MoPH and central government’s enabling legal and policy environment (+)
Geography	• Accessibility of health services to population (+/-) • Willingness of health professionals to serve in remote/insecure areas (+/-) • Ease of access for supplies (+/-) • Ease of access for monitoring (+/-)

### Sociocultural norms

Sociocultural norms at provincial level were identified by all categories of participants, from policy makers at the MoPH to donors to provincial- and field-level health workers, as a key factor influencing the delivery of health services [MoPH-02][DPR-01][HW-07][PPHD-07]. For example, in some provinces it is culturally unacceptable for a male health worker to examine a female patient. Coupled with a relative lack of women with higher education, leading to a shortage of local female health workers, this situation compromises health care access for women. In other provinces, different sociocultural rules about female modesty and gender apply. In these provinces, socio-cultural norms allow women to be examined by male health workers and as a result, women have better access to health care regardless of educational level. For instance, in Bamyan women actively participate in the health care system, which is functioning. Women provide some health services, and female and male health workers working together is the norm. In other parts of the country, such as Nooristan, this would not be considered culturally acceptable, requiring a different health system structure [MoPH-02].

### Political and security factors

Successful leaders are marked by their ability to maintain close relationships with local people and agencies [24]. The level of, capacity at, and structure of, the provincial health department was mentioned by participants as a key element affecting the delivery of health services in general, and contracted-out services in particular. Participants’ views were similar at the central and provincial levels. For instance, policy makers in Kabul felt that a provincial health director could facilitate better provision of health services by NSPs by making resources available and promoting the success of health service delivery efforts [MoPH-02]. The capacity and structure of the provincial health department is linked to other provincial leaders such as political figures, influential local residents and the provincial governor. However, all participants described unwanted political interference in decisions related to the delivery of health services, such as choosing where to deliver services, pushing for the hiring and firing of certain health workers or contracting with specific companies for logistical support. One respondent stated:

Sometimes the politicians interfere [with the implementation of the health services]. They recommend to the NGO an irrational establishment of a health center. [As a result] underutilized clinics are created due to political reasons. The CHC [Comprehensive Health Center] is established but population around it is not sufficient [to reach the targeted number of clients]. [DPR-01]

Political interference has proven a key challenge to contracting out programs, as NSPs have to work with local officials, warlords, members of Parliament and other influential members of the community on a regular basis. [MGR-03].

Provision of services by NSPs was also considered a challenge to government authority. Several provincial government officials interviewed reported that the CO mechanism has undermined the role of the government in service provision, consequently calling into question the legitimacy of the government. Government officials expressed concern that the population only perceives that the services are provided by NSPs and does not understand the government’s role in providing health care services [PPHD-06] [PPHD-04].

Respondents in all categories unanimously stressed that security is an essential factor in creating an enabling environment for the effective provision of health services by NSPs. Respondents from Nangarhar and Kandahar expressed the most concern about security. Insecurity is debilitating to the delivery of health care. Several interviewees described the impact of worsening security in some provinces after 2007. Increasing insecurity in these areas affected both the delivery of services and reduced the ability of the MoPH to conduct monitoring and supervision [MoPH-02]. Those NSPs with long-established relationships with local communities have generally managed to continue delivering services even in areas controlled by anti-government groups, although many incidents were mentioned when clinics had to close, or were even attacked, during a local conflict. A respondent from one of the least secure areas summarized the problem:

War, and the local situation, have huge impact on [health services]. If somewhere there is war and the situation is not normal then an NGO can’t find qualified staff and can’t provide [health services]. [PPHD-06]

### Geography

Geographical features also have a significant impact on the reach and effectiveness of health services. Each province of Afghanistan has distinct geographical characteristics that affect the distribution of health facilities and provision of health services. It is particularly difficult to guarantee regular supplies of medicines and medical equipment in hard-to-reach areas in mountainous regions. The difficult geography is compounded by challenges created by the climate. NSPs have to plan ahead to maintain services during often long periods of road closures in winter.

Many NSPs, therefore, prefer to provide services in regions that are easier to access. NSPs with contracts to deliver health services in regions with harsh geography need to develop innovative strategies, in particular to incentivize recruitment and retention of health professionals willing to work in difficult conditions. Several participants from NSPs mentioned instances when they had to offer more benefits to get staff to accept positions in hard-to-reach areas. This was particularly the case for female doctors, whose packages might include also hiring the doctor’s husband, providing hardship payments and offering special vacation opportunities [PMGR-03]. One participant described that:

NGOs’ salary rates are according to geographical grading. It’s different in different provinces. The hard-to-reach areas and conflict-affected areas have more salary. [HW-303]

Contextual factors lay the foundation on which institutional responses are built and in terms of which the contractual factors are defined.

### Contractual factors

The category of contractual factors includes various aspects of the contracting mechanism: types of services covered, contract formality, contract duration, contractor selection, specifications of requirements, processes of contract implementation, output and outcome indicators and finally the contract payment mechanism.

### Types of services

Respondents were generally able to describe the main types of services contracted out by the government to NSPs (including BPHS and the Essential Package of Hospital Services (EPHS)) and those provided directly such as provincial hospital services. One focus group agreed:

[The] Basic Package of Health Services provided in [our] province includes all the BPHS components, such as maternal and neonatal health services, immunization and child health services, public nutrition, control of communicable diseases services, mental health and disability health services, and pharmaceutical services. There is also EPHS, which provides secondary health services through the regional hospital in [a neighboring] province. The EPHS is a contracting-in mechanism in [our] province. [FGD-02]

Some respondents also mentioned contracting of capacity building programs and research projects.

### Contract formality

MoPH policy makers expressed generally favorable perspectives on the contracts in the CO mechanism. Several respondents mentioned that the selection process established for CO and the creation of the Grants and Contracts Management Unit (GCMU) have become examples that other national sectors seek to follow [MoPH-02] [PPHD-02]. From the outset, the BPHS program has emphasized formality in its contracts. They require NSPs to abide by all governmental laws (after undergoing a rigorous selection process). These strictures enabled the NSPs and the government alike to trust each other, and fostered reliability of the services.

### Contract duration

Both NSP managers and MoPH officials interviewed noted that contract durations differed by donor, and that contracts were commonly extended beyond the original contractual agreement. While the initial contract durations ranged from 18 to 36 months (with an average of 26 months), extensions lengthened them. One MoPH official explained:

[The durations] are different, normally between two and three years. But these [contracts] were extended. Even if it is for three years, it is subject to the evaluation by the [third party] organizations. Performance review is a condition for the extension. There were extensions up to five years. The PCH [partnership contracts for health], for instance, started in 2009 and in the second year it was evaluated and extended to the third, and finally it was extended to five years. The small project [non-BPHS] did not last more than six or seven months. [MOPH-02]

Respondents had mixed reactions towards the extensions. Some argued that the extensions of contracts had a positive effect on service delivery by preventing disruptions that would occur with another long tendering process. This view was supported by NSPs, who stressed that the longer an NSP worked in a given location, the stronger their relationship with the community [PMGR-02]. However, others presented a different viewpoint. This view was widely expressed by provincial MoPH authorities, who reported that following an extension the NSPs tended to relax, undertaking fewer quality improvement efforts or innovations [PPHD-05]. Another concern raised about contract extensions was that they reduce competition, undermining its benefits.

### Contractor selection and parties to the contracts

Funding for the contracts comes from multiple donors with the MoPH now serving as the purchaser; in the earlier stages of the CO program, donors interacted directly with NSPs believing that the government lacked the requisite capacity for financial management and procurement. Indeed, some donors temporarily used their own mechanisms for procurement of NSPs until the government’s procurement capacity was ready to manage a large program like the BPHS [PPHD-01]. Once the MoPH had developed the capacity to handle procurement for large scale programs, a unified system was developed with the leadership of the MoPH. This transition occurred gradually, beginning with the 2003 transfer to GCMU of contract management for all the World Bank funded provinces. In 2010 USAID delegated its contract management to the MoPH, as did the EU in 2013 [5].

The study reviewed the contract specifications from the MoPH. According to these documents, the process for contracting out to NSPs is well-designed and clear. The process is governed by the MoPH with active participation from relevant stakeholders, including provincial health directors. A selection committee (GCMU officers, provincial health director, UN agency representative and MoF representative) reviews and awards contracts, while the administrative aspects are managed by GCMU.

While the process seemed clear on paper, interviewees expressed concerns. Some respondents suggested that there was little real competition. Some felt that the participation of provincial health directors was merely symbolic; moreover, MoPH officials at both central and provincial levels expressed concern that a small number of Provincial Public Health Directors (PPHDs) were unable to be impartial.

### Specification of contract requirements

BPHS and EPHS documents specify the services to be provided by NSPs. They detail the requirements for all processes, inputs and monitoring, as well as targets for outputs and outcomes. Among our respondents, MoPH managers, donors and central NSP managers had more precise knowledge of these details than health workers and provincial managers.

### Implementation of contracted services

The process for implementing health services is specified by the BPHS implementation guidelines. Each contract includes a log frame and approved and agreed-on indicators that help guide implementation and monitoring and evaluation of the performance of NSPs. Thus there is a common understanding between the government purchaser (MoPH) and the NSP contractors about what types of services are to be provided and how they should be implemented [MoPH-03].

In this study, all groups of respondents demonstrated high levels of awareness of performance specifications and most discussed performance indicators. The responses from NSP employees in particular showed that these indicators play a meaningful role in ensuring that services are delivered per the plans and expectations of the contracts [PMGR-01],[PMGR-02],[PMGR-03],[PMGR-04],[PMGR-05],[PMGR-06].

### Output and outcome indicators

Each contract includes specific and clear target output and outcome indicators. These contribute to transparency and clarity on how to measure activities and facilitate quantification of the services provided by NSPs. Output indicators may include number of health workers trained, number of health education sessions conducted or number of institutional deliveries. Output targets are based on the population of a basic health center (BHC) or clinic catchment area. Provincial targets are set using provincial population data. Outcome indicators are captured and measured separately by third party evaluators using the Balanced Score Card (BSC). The BSC has six domains [25]. (Additional file 1 provides information on BSC performance over time for each of the six provinces examined in this study).

Outputs are the primary focus for USAID-funded contracts, which reimburse NSPs for services delivered. This payment system facilitates evaluation, as data are reported. The World Bank -contracts and the current System Enhancement for Health Actions in Transition (SEHAT) program, on the other hand, are based on lump-sum contracts and emphasize outcome indicators. One Ministry official explained:

The three donors have had different performance indicators. For USAID, input process and output and outcome indicators were used. We had a datasheet that contained both output and outcome indicators. The World Bank had more focus on outcome indicators and did not emphasize process or inputs. EU was in between, with a tendency towards outcomes. [MOPH-02]

### Contract payment mechanism

As noted, two mechanisms have been used to pay contracted NSPs: lump-sum payment and cost-reimbursable payment. The World Bank funded projects were contracts with a lump-sum payment mechanism, as one respondent described:

The contract was lump-sum, with some flexibility in movement across the budget lines. The staff is provided with salary and money for some other items, such as running cost, maintenance and emergency medicine. [PMGR-04]

The cost-reimbursable payment mechanism, on the other hand, is the main model under USAID. In USAID-supported provinces, payments were made based on reported outputs.

The EU contracts fell between the two distinct models. They were cost-reimbursable, but with a greater focus on performance outcomes rather than inputs and outputs.

NSPs managers we interviewed expressed preferences for the lump-sum mechanism, which they see as offering more flexibility and less rigorous reporting and monitoring [PMGR-02, FGD-01]. However, this mechanism risks making evaluation using reported data more difficult. Respondents from the government, therefore, generally preferred a reimbursable mechanism, which entails more scrutiny and closer supervision of the NSPs [MOPH-02, PPHD-05].

The choice of payment mechanism can affect performance. With lump-sum payments, NSPs have more freedom in terms of their implementation processes. They have latitude to initiate innovative approaches to attain the contractually agreed upon outcomes. However, it also creates more opportunities to diverge from the contract.

With the launch of the SEHAT program (2013), however, all payment mechanisms are lump-sum. However, “lump-sum” may mean different things to different partners. One respondent highlighted this conundrum:

Everyone talks about lump-sum mechanism but still there is not enough clarity about it. NGOs have their own definition where they want more freedom and flexibility, while MoPH has its own definition trying to make NGOs more accountable. Both parties should come together and decide what they mean. [MoPH-02]

Frontline health workers understood “payment mechanism” in reference to their salaries, regardless of the contract model used to support the payroll. One provincial worker described:

The payment mechanism for the employees is working in such a way that first the reports from the health facilities are collected by the NGO. Then, the reports are analyzed and the financial report is prepared and finally, the employee payment is deposited into their bank accounts on a monthly basis. In the past, this payment mechanism was different. The staff payments were processed in the form of a cash transfer.

The payment systems for employees have evolved. In the first few years, NSPs determined salaries based on their budgets. In 2005, a national salary scale was established by the MoPH that standardized payments across the provinces and organizations. Most health workers interviewed thought that a Results-Based Financing (RBF) approach would be more appealing than a fixed salary, because they would get both a basic standard salary and extra payment based on performance [HW-05].

The contractual factors establish parameters for how contractors respond to contextual factors, and set limits within which the institutional factors operate.

### Institutional factors

We classified institutional factors in two categories: *internal* responses (created by either the purchaser or the contractor) and *external* responses [21]. *Internal* responses are further divided into three sub-categories: 1) managing inputs, 2) managing outputs and outcomes, 3) performance monitoring. *External* response sub-categories are: 1) provider market and 2) public service.

### Internal institutional factors

#### Managing inputs, outputs and outcomes

These factors address NSPs’ various approaches to using the inputs they receive under the contract to implement health services. Human resources management, our respondents reported, is a pivotal and highly challenging aspect of contract management for NSPs [MoPH-02, MoPH-03, PMGR-01, PMGR-02]. While national regulations and contract specifications exist to regulate hiring (and firing) of staff employed under the contract, some flexibility exists and further exceptions can be made. This enables NSPs to avoid lengthy government human resource management procedures, resulting in more efficient provision of quality health services. The contracts oblige NSPs to provide a list of key staff to the MoPH in advance; field officers and health workers must be recruited as soon as possible once the project starts. NSPs are responsible for filling vacancies and planning coverage for staff vacations [PMGR-01].

Health workers’ commitment to the project has been a persistent challenge. Despite the fact that the number of health workers trained has increased exponentially in all categories (doctors, nurses, midwives and others) since 2003, the country continues to face a shortage of health human resources. NSPs are authorized under their contracts to offer relatively high salaries based on the National Salary Policy; however, the rate of staff turnover was high in some provinces. As mentioned, finding women to fill key field positions proved particularly challenging for NSPs [FGD-01].

NSPs described effective and innovative responses to human resource management issues. One effective strategy was to hire staff from neighboring countries to be deployed in Afghanistan. On other occasions, NSPs consulted with the MoPH to create attractive payment packages for serving in difficult to reach areas [PMGR-04, HW-10].

Equipment and medical supplies are also critical inputs. However, these were less frequently discussed in our interviews. The importance of on-time and regular supply was noted, as was the key challenge with equipment: maintenance. Although biomedical engineers and companies with post-purchase services are present in Kabul, they generally unavailable outside the capital city. Instruments that break down are not repaired in a timely way, leaving health care providers without important tools. As mentioned in the geography factors, health centers located in hard-to-reach terrains also face seasonal challenges, as NSPs must receive sufficient medical and pharmaceutical supplies to last through the winter [HW-201] [MGR-01].

Pharmaceuticals are vital inputs to health services. The availability of medicines in a health facility is one key indicator of functionality; stock-outs limit effectiveness of health services and undermine patient satisfaction. Respondents reported that the purchase of medicines is a critical issue in the provision of inputs for NSPs. Two mechanisms were used for purchasing medicines. One is the centralized purchasing system recommended under USAID grants. In USAID-funded provinces, medicines were procured from internationally accredited companies by Management Sciences for Health (MSH) or another organization, and distributed to provinces in response to requests from NSPs. This model emphasizes ensuring quality of medicines. The alternative model is a decentralized mechanism that provides NSPs with funds to purchase medicines directly from certified pharmaceutical companies according to criteria provided by the MoPH. This model provides more flexibility for NSPs and reduces the risk of stock-outs [MoPH-02].

Since all provinces were brought under the SEHAT project, all medicines purchases are now decentralized. One respondent, however, felt that the most efficacious mechanism still needs to be determined. While the various donors had different preferences regarding purchasing, representatives of NSPs indicated that they prefer the decentralized system because it allows them to procure pharmaceuticals from the local market on a regular basis [PMGR-09].

Infrastructure is another input that affects the effective provision of services. Because the construction of new health centers is expensive, it is generally not included in NSPs’ proposals. This situation originates from two flaws in the contracts’ legal framework. First and foremost, NSPs seek to minimize costs to reduce the total budget of their proposals to make them more attractive bids. Second, the procurement policies of both the government and donors discourage infrastructure development. However, in 2003, the USAID provided funding to construct a large number of health facilities across the country. Where government facilities are not available to serve as health centers, some NSPs rent local houses or other buildings and convert them into health facilities. This, according to some respondents, is the most common practice for swift start up.

### Performance monitoring

Our interviews found that most stakeholders have a positive impression of performance monitoring for contracting-out health services. A national HMIS system and third-party evaluations are included in the contracts to track input, output and outcome measures, as well as to assess overall impact.

The HMIS is based on a set of indicators gathered at the health facility level by frontline health workers, such as the number of deliveries that occurred in the health centers or were assisted by skilled birth attendants and the number of children vaccinated through routine immunizations. However, since the HMIS data are based on self-reports from providers, their quality and accuracy were called into question by some respondents. The new system for HMIS data verification, which involves a third party, received positive feedback from some respondents, who indicated that it is helping to improve the reliability of HMIS data [DPR-02, FGD-01].

A second concern with HMIS data is its usefulness for decision making. Some respondents mentioned that HMIS data are indeed informing decision making at different levels, from the individual health facility to the ministerial level. One policy area in which HMIS data is considered to be highly valuable is in the rationalization of distribution of health facilities. HMIS data provide information to help assess whether, considering both the investment costs and the needs of communities, proposed locations or functionality levels of new health centers are rational.

Respondents reported that NSPs have also created systems to utilize collected data in improving the delivery of health services at different levels. Data collected from clinics are analyzed and presented back to health facility managers on monthly and quarterly basis. Any indicators that have not been achieved are highlighted and corrective measures discussed. For instance, if the number of deliveries in a facility is low, the NSP conducts a follow-up assessment to understand why. This informs decision making on how to address problems so corrective measures can be integrated in the plans for the next cycle.

In summary, the MoPH in collaboration with donors and its development partners has established a comprehensive, intensive and responsive HMIS to measure and provide timely feedback on the contracted NSPs’ performance on indicators. Some concerns remain about the quality of the data and the efficiency of monitoring and evaluation (M&E) processes. However, on the whole the system covers all aspects of the project and is well integrated, thus constituting the backbone of CO for health services.

#### External (provider market) responses to the scheme

The CO approach to health service delivery has affected three provider types: not-for-profit NSPs, for-profit NSPs and the government. Because health services have been contracted out only to not-for-profit organizations thus far, the first category is discussed in more detail than the other two.

#### Not-for-profit NSPs

Most of the interviewees agreed that CO has improved competition and quality among NSPs delivering health services in Afghanistan. Previously, each NSP had its own donors and catchment areas, and they paid little attention to competing with each other. The advent of the CO process revolutionized the provider market and drastically changed the context. NSPs now had the opportunity to apply for BPHS contracts for specific locations and periods of time, while the funding from all donors was aggregated in one basket fund and channeled through one bidding mechanism.

One positive outcome of the shift to CO has been the provision of growth opportunities to new and local NSPs. Local NSPs are increasingly winning bids, as one respondent described:

For example, in the beginning [before the start of outsourcing health services], there were few organizations in the health sector [with the capacity] to manage health facilities, but now by contracting out there are many local NGOs who could properly manage around 90 health facilities at a time. [PMGR-06]

Our study revealed two perspectives on the roles of NSPs in Afghanistan. One perspective expressed by NSP managers and some MoPH officials focused on the positive outcomes and impact of health services delivered. In contrast, however, some MoPH provincial staff expressed antagonism towards NSPs, referring to cases when NSPs did not fulfill their requirements effectively or efficiently [PPHD-05].

Thus while some see the increase in the number of NSPs as a positive outcome, others remain skeptical and concerned about having too many NSPs in the market. The debate is currently of paramount significance, as local public health departments have begun arguing that the government should contract with the public health directorates at the sub-national level, instead of NSPs, for service delivery. At the same time, debate is occurring at the cabinet level regarding the merits of the CO process and the option to switch to a contract-in mechanism [FGD-01]. One interviewee expressed reservations about the motivations of some involved in the debate:

I have a concern about PPHDs. Although PPHDs are the owners of the projects, they have a negative competition with NGOs [and] they are dissatisfied all the time and show jealousy towards NSPs because they [PPHDs] could not implement such projects. [PMGR-09]

Other respondents expressed their opinion that provincial-level teams should focus on their roles as regulatory and enforcement bodies, providing leadership and monitoring for BPHS programs rather than implementation.

#### For-profit NSPs

BPHS has so far never been contracted out to a for-profit company or organization, although there is no regulation against it. The for-profit private sector market has been affected nevertheless by CO of NSPs. Some respondents suggested that for-profit companies have been restricted to providing secondary and tertiary health services in urban settings because they cannot compete with government-supported primary health centers in rural areas:

In my province, the for-profit organizations could not grow because most of the services are provided by health centers supported by the government and as a result, there is no place for them. [PPHD-5]

As a result, for-profit health centers remain weak in provision of primary health services. Other respondents felt, however, that the private sector has grown stronger where NSPs failed to provide quality health services. In these areas, patients seek services from the for-profit private sector when they are not well cared for or not satisfied at primary health centers [PPHD-06].

#### Government’s response

The impact of the CO program on the Afghan government’s capacity and service delivery arrangements were evaluated positively by respondents. Interviewees highlighted two aspects. First, they stated that the program has helped the MoPH prove itself to be a public agency capable of managing large projects at the national level. Second, respondents pointed to improvements made in government capacity to conduct procurement and financial management [FGD-01]. These capacities will enable the government to continue implementing services into the future, as one respondent described:

Contracting-out mechanism had its positive impact at the level of MoPH: its capacity improved in contract management. This system encourages the government to improve its capacity to implement [something] such [as] this project. [PMGR-04]

Some respondents also described how CO has boosted the economy by providing capacity-building opportunities to health workers, creating jobs, supporting local pharmaceutical and medical supply markets, and encouraging competition among providers. Whether the government can and should itself become a competitor, providing health services is still under evaluation. It could be a good option in the long run, but for now the MoPH is successfully supporting NSPs to provide health services [MoPH-03].

## Discussion

The present study offers a theoretically sound and in-depth qualitative exploration of the contextual, contractual and institutional factors that affect the implementation of contracting out health services to NSPs. These factors form the key elements of a framework used frequently for evaluating contracting of health services [21]. The framework suggests that interactions among the many factors in the framework can result in better health care delivery, which in turn improves health impact. This study also did not look at health impact directly; however, it projects that the collective impact of these and possibly other factors have had positive impacts on health in the regions of Afghanistan receiving CO services. Maternal mortality and child mortality rates improved considerably from 2003 to 2013. The Afghanistan Mortality Survey (AMS), conducted in 2010, also showed improvements in the overall health of the population compared to the data from a survey in 2002 [8, 26].

Our findings on how contextual factors affect the contract out process are aligned with others’ findings. Mills proposed that the social, economic and political environment can facilitate or restrict a successful CO program [27]. For example, if the legal system, banking system and government procedures are weak, contracting will be difficult [27]. Another study proposed that the state and private sectors can play an important role in creating a conducive environment for smooth implementation of contracted-out services [27]. Our study followed Liu et al. by categorizing contextual factors into political, geographical, and economic and sociocultural factors in the external environment [21]. We expanded the external environment to also include climate and security concerns; we recommend that other researchers applying the Liu et al. framework in a post-conflict and/or fragile state also expand their focus to include these or other relevant contextual determinants.

The health care delivery program in Afghanistan was designed to promote equity, focusing on reaching poor people and individuals living in remote areas with health services. However, we found that insecurity (including risk and fear of violence, being killed or kidnapped, and the presence of armed conflict in general) was one of the main factors adversely affecting the CO health services. Similar trends are reported elsewhere. For example, a study of post-conflict health reform in Uganda enumerated insecurity and lack of institutional capacity as predominant factors affecting the process of building up the health system [28]. Newbrander, Waldman and Sheperd-Banigan emphasized security as a critical determinant for a successful contract-out program [29]. These authors also point out that conflict areas may require different types of health services from peaceful areas. Our study supports this: the full package of health services has been provided in more secure provinces in Afghanistan, while insecure areas may only receive emergency services.

In Afghanistan, NSPs were needed to support the urgent delivery of health services that the government was not in a position to provide. The legal framework in Afghanistan, paired with support from the government, enabled the initiation and implementation of contracting NSPs [30], although resistance and tension at the outset of the CO scheme were reported. Newbrander et al. reported that some NSPs were concerned about maintaining their independence [30]; another tension comes from the concern that there is a dichotomy between state-building and delivery of services through NSPs [30].

Institutional factors, such as management of human resources, also influence the success of CO. Newbrander et al. described human resource management as a central aspect of contracting out [3, 5, 9]. They suggested that to improve human resources requires establishing collaborations with training institutions and transitioning towards certification/accreditation programs [10]. The shortage of health workers in all categories was reported as a key challenge in our study; however, contracted NSPs have coordinated with the MoPH to identify innovative solutions. Some proved more successful than others—finding female health workers willing to serve in hardship posts remains a significant challenge, as is the supply of pharmaceuticals. The shortage of female health workers has also been described by the MoPH and others [3, 5, 9, 31].

NSPs and the MoPH have also collaborated to address challenges with other institutional factors such as procurement mechanisms. Stock-outs and low-quality medicines at facilities reduce patient satisfaction and can lead to declines in outpatient visits. Purchasing from local markets through a decentralized mechanism improves the availability of medicines but may undermine quality.

Study participants extensively discussed the institutional approaches to performance monitoring, noting that a significant amount of energy and resources are invested in measuring progress of contracted programs. M&E of the performance of NSPs contributes to accountability and the effective provision of services. The government emphasizes close monitoring of inputs, outputs and outcomes of health services contracted out to NSPs; NSPs have complied with these requirements. At central and provincial levels, the MoPH utilizes various monitoring mechanisms through its M&E department, the HMIS program and GCMU administrative procedures. Independent evaluations conducted by external organizations and based on BSCs are another hallmark of the CO program. NSPs have developed their own M&E systems to comply with their contractual requirements [11]. Edward et al. emphasized the pivotal role of BSCs in improving transparency, governance and NSP performance benchmarking [32]. The important contributions of the HMIS in monitoring NSPs’ performance have also been emphasized by numerous authors over the past decade [3, 5, 9, 31].

Outside the CO scheme, the health care provider market has been affected by contracting out health services to NSPs. CO created new opportunities and competition on quality and cost of services among the not-for-profit NSPs bidding to provide BPHS and EPHS services. International NSPs have increasingly been underbid by local NSPs, whose administrative and overhead costs are lower. The impact on for-profit health care providers seems mixed. Contracting-out reduced the market share of for-profit organizations providing primary health services, but private clinics and hospitals reportedly remain effective in providing specialized medical services. Contracting out has, as yet, changed little for the government as a health care provider. Except in three provinces, the government is not competing with NSPs to provide primary care.

Liu et al. proposed that contracting out has an impact on contestability in the provider market, improving the environment for competition among providers [21]. Our findings concur with this in the case of the not-for-profit NSPs providing primary health care. For-profit organizations, on the other hand, focus on secondary and tertiary health services [21, 33]. We suggest further research be undertaken to understand how to better involve the private for-profit sector in the provision of primary health services.

Key recommendations to policy makers for addressing all three sets of factors are presented in Table 5.

**Table 5:** Recommendations derived from study findings

Recommendations on Institutional Factors	
Contract Specification	Hire a third-party to conduct evaluation of the intended outcomes
Contract Formality	• Include clear selection criteria • Establish a unit/mechanism to ensure that the criteria are enforced
Payment Mechanism	Install a unified and homogenous payment mechanism at the outset
Recommendations on Contextual Factors	
Political Context	• Foster political will for initiating and enforcing contracting out – this is the single most important contextual factor • Ensure that political support and an appropriate legal framework exists • Develop mechanisms to limit inappropriate interference by local government leaders
Geographical Context	Establish a contracting out system that acknowledges, respects and addresses geographical variations and relevant adaptations
Security Context	For a country in a conflict or post-conflict situation: • Ensure that NSPs fully understand the risks of service provision in insecure areas and the difficulties likely to arise • Establish direct and clear communication with all partners and stakeholders on all sides of the conflict
Recommendations on Institutional Factors	
Internal Response: Input, output and outcome management	• Explore innovative approaches to recruitment of female health workers to address access issues • Improve pharmaceutical procurement management and monitoring to avoid stock-out and low-quality medicines • Focus on making observable change in the health of communities. Enhance patient satisfaction by monitoring behavior of health workers and managers
Internal Response: Performance monitoring	• Use multiple triangulation methods to assure quality of data • Establish a single department and system responsible for all performance monitoring • Align monitoring and evaluation mechanisms among NSPs, government and donors
External Response: Provider market	• Develop and implement policies that prevent a few large organizations from monopolizing health care delivery • Encourage economies of scale by coordinating multiple contracts to any individual NSP • Identify strategies to engage the for-profit sector in the provision of health services
Overall	• Consider multiple factors when contracting out to NSPs • Recognize that a universal BPHS policy might not be appropriate across the country; province-specific criteria could strengthen implementation

### Limitations

Liu et al. note that systematically understanding the interaction of factors requires comparators [8]; this was beyond the scope of this individual country level analysis. Other limitations related to three aspects of the research process. The study design focused on collecting and analyzing qualitative data to generate an in-depth picture of the contracted health care delivery system in Afghanistan. However, the findings could also have been triangulated with quantitative data, in particular to understand the CO program’s outcomes.

Execution was limited by insecurity, the geographic size of the catchment areas and difficulties posed by transportation. Further, given time and resource limitations, the qualitative research design used purposive sampling of provinces and participants in order to capture a breadth of experiences in terms of payment mechanisms, contracting processes and KIs’ roles. However, we cannot make claims about how common or widespread any of the perspectives were. During data collection, we faced particular challenges when interviewing PPHDs. In some cases, they lacked institutional memory about contracting out, while others were not reachable. In an exceptional case, one director of health was interviewed while hospitalized and recovering from a roadside explosion.

Finally, our main objective in this study was to present a description of the factors influencing a specific intervention. However, analyzing interactions among the factors proved beyond of the scope of this study. Future studies are recommended to delve further into this.

Our relatively narrow case study on the BPHS allowed us an in-depth view of the factors that affect NSPs’ performance. We omitted discussion of the contracting-out of EPHS or other programmatic, training and research services. We sought to highlight this gap by mentioning them in the background section, and recognize that they present areas for additional research.

## Conclusion

Contracting-out to NSPs to provide the BPHS has been a successful strategy in Afghanistan that is influenced by many factors. We recommend that the MoPH considers various factors beyond the BPHS specifications when developing contracts to deploy NSPs. In particular, a universal BPHS policy may not work equally well in all provinces. Province-specific criteria for selecting and contracting NSPs could strengthen BPHS implementation. In addition, awarding multiple contracts to a single NSP may lead to a monopoly, resulting in inefficiency. We recommend that the MoPH explores engaging with the private for-profit and government sectors for BPHS service provision in order to engage a wider range of stakeholders, with their own innovative and creative approaches, to reach all Afghan citizens with accessible quality primary health care services.

## Additional file

Additional file 1: Health systems performance by province. (DOCX 759 kb)

## Abbreviations

AHSPR: Alliance for Health Systems Policy and Research
AMS: Afghanistan Mortality Survey
BHC: Basic Health Center
BPHS: Basic Package of Health Services
BSC: Balanced Scorecard
CHC: Comprehensive Health Center
CHW: Community Health Worker
CI: Contract In
CO: Contract Out
COI: Co-Investigator
EPHS: Essential Package of Hospital Services
EPI: Expanded program of immunization
ERC: Ethical Review Committee
EU: European Union
FGD: Focus Group Discussion
FI: Field Investigator
GCMU: Grant and Contract Management Unit
GLICS: Global Innovations Consultancy Services
HMIS: Health Management Information System
IIHMR: Indian Institute for Health Management Research
IRB: Institutional Review Board
JHU: Johns Hopkins University
KII: Key Informant Interview
M&E: Monitoring and Evaluation
MoPH: Ministry of Public Health
MSH: Management Sciences for Health
NGO: Non-Governmental Organization
NSP: Non-State Provider
PBI: Performance-Based Initiative
PI: Primary Investigator
PPHD: Provincial Public Health Director
RBF: Results-Based Financing
RC: Research Coordinator
SEHAT: System Enhancement for Health Actions in Transition
USAID: United States Agency for International Development
WB: The World Bank
WHO: World Health Organization
