# Supplementary material for: Factors influencing performance by contracted non-state providers implementing a basic package of health services in Afghanistan
Source: Int J Equity Health. 2018 Oct 5;17:128. doi: 10.1186/s12939-018-0847-4 (PMC6172740; doi:10.1186/s12939-018-0847-4)
Supplement: Supplementary file 1 — Health systems performance by province. (DOCX 759 kb) [file 12939_2018_847_MOESM1_ESM.docx]

## Additional File 1 : Health systems performance and functional NSPs by province

We used Balanced Scorecard reports from 2004 to 2013 to review how the NSPs were performing in the selected provinces. Kabul, Bamyan and Balkh showed overall lower performance while Herat, Nangarhar and Kandahar has shown a higher overall performance. To better understand the provincial profiles and how the performance of the NSPs have fluctuated it is important to understand the concept of overall provincial performance means which is in fact the arithmetic means for all 28 indicators and six domains of the Balanced Score Card that is available for every province and every year since 2004. In the coming sections, we use this indicator to show the performance of the selected provinces.

### Kabul

From 2003 to 2006 in Kabul, province health services have been contracted out to four different NSPs. Since it is rather a large province, MOPH decided to divide it into three clusters and each cluster was contracted to a specific NSP. These NSPs are Bangladesh Rural Advancement Committee (BRAC) for C1, STEP Health and Development Organization (STEP) for C2, and IMC C3. From 2006 to 2009 the services were again contracted to STEP. The donor for the health services has always been USAID. In the third cycle (2009-2015) the entire province was contracted out to BRAC.

Kabul has been relative secure from 2004 to 2013. The insecurity issues in Kabul city and adjunct districts were limited to suicidal bomb explosions and sporadic incidences. Kabul city is an urban area and is running through strengthening mechanism (SM) funding by WB. Recently some of the key clinics in Kabul city have been contracted to certain organizations (e.g. Noor Mohamad Shah Meena Clinic is being run by Medicine Sans Frontier [MSF]).


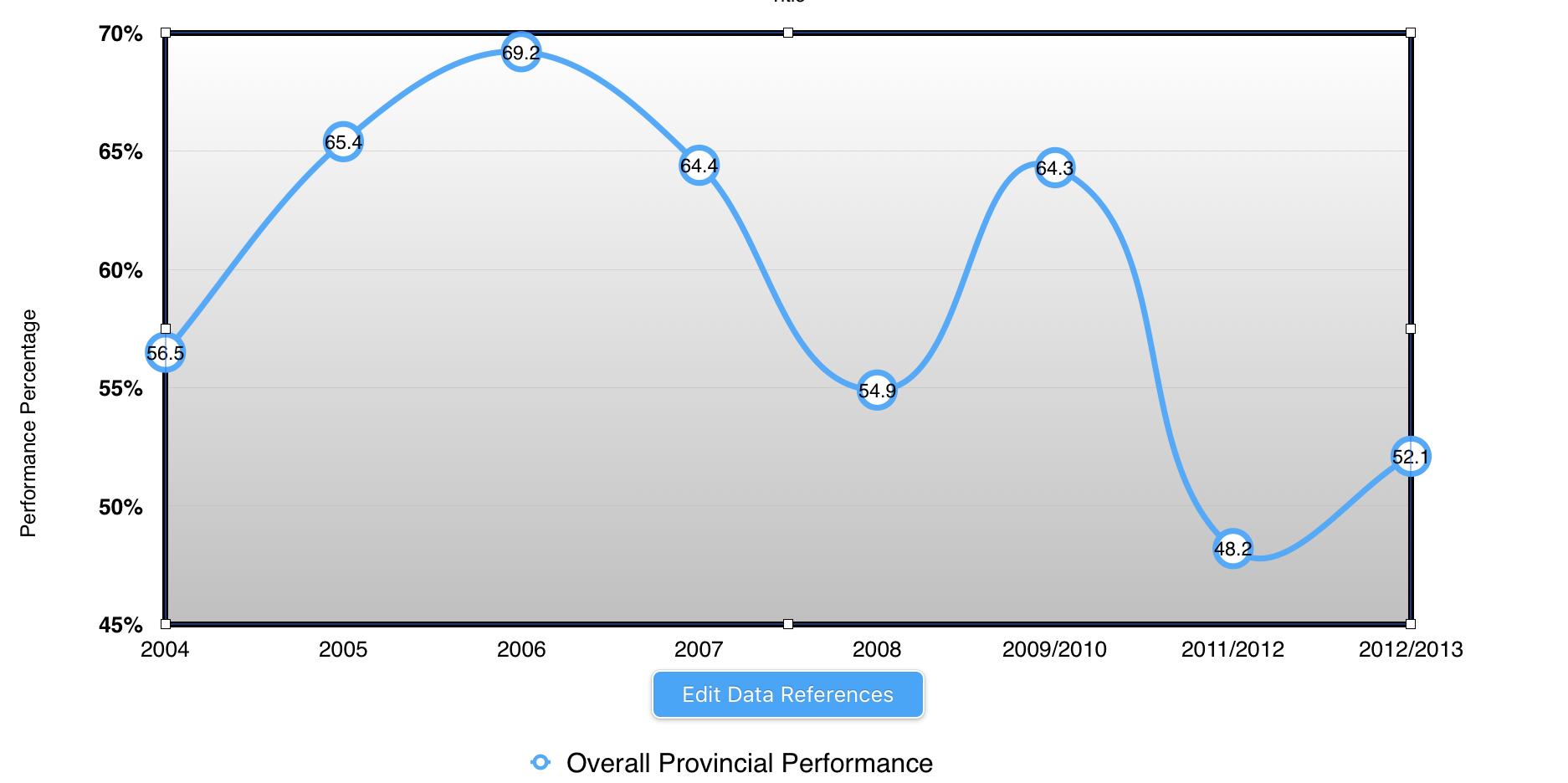


**Years**

Chart 1

Kabul BSC Means Score Differences (Source BSC Reports)

Geographically Kabul is a combination of mountains and valleys. It does not have a harsh winter and its summers are not that hot to affect the health service implementation. As a result, there are no barriers in terms of transportation. Kabul has 15 districts and in the first cycle, it was clustered into three clusters. Since Kabul districts are close to the Capital they benefit from the high presence of security forces which have a heavy presence but at the same time, these areas suffer the most because insurgents use these areas to enter the city or create disruptions. This paradox insecurity has affected the health services.

The percentage of a performance difference in lower benchmark has reduced 45.5 % between 2004 to 2013 (from 100% to 54.5%). The mean score has reduced 4.4% (from 56.5 to 52.1). See chart 1.

In Chart.1 we read that the Balanced Scorecard results for Kabul had a variable performance with a zigzag curve, first it was low (2004-2005) and then it improved (2006) subsequently it fell (2007-2008) with a decline in coming years (2009-2010) eventually decreasing (2011/2012) to a status lower than it started.

Most of the indices were in the YELLOW in 2004 with two indexes in GREEN and no RED. From 2005 to 2009 all domains and indexes have shown great results mostly GREEN. In 2011/2012 Kabul had more indexes in RED than ever before with a means score of 48.2.

Since 2013 we see a negative trend in some of the domains such as physical capacity and quality of services. The Balance scored card of 2012-2013 indicates that Kabul has risen from 32th to 29th place. This rise is more evident with the following indicators:  Salary Payment Current 7. Provider Knowledge Score 11. Laboratory Functionality Index (CHCs only), Functional Infrastructure Index, Client Background and Physical Assessment Index, Client Counseling Index and Health Facility Management Functionality Index ^30^

### Balkh

In Balkh province health services have been contracted out to BRAC/BDN (2004-2009) and CHA (2010-2013). The donor for the health services has always been the World Bank. The security has been good all of those years. Except for few incidences here and there Balkh province has proved to be a secure place for another Afghanistan so the contract out services have not been affected by it.

The Balanced Scorecard of 2004 shows that the performance in this province has been most of its benchmarks in both upper and lower levels (BSC￼). In 2008 Balkh fell behind (means score of 68.8 across all 26 indicators). The patient satisfaction indicator and proper sharp disposal were among the key indicators contributing to this fall back. With all these in 2008 Balk remained in the GREEN. In the following years Balkh fell back to the YELLOW areas. (Please see Chart 2 for graphic representation)


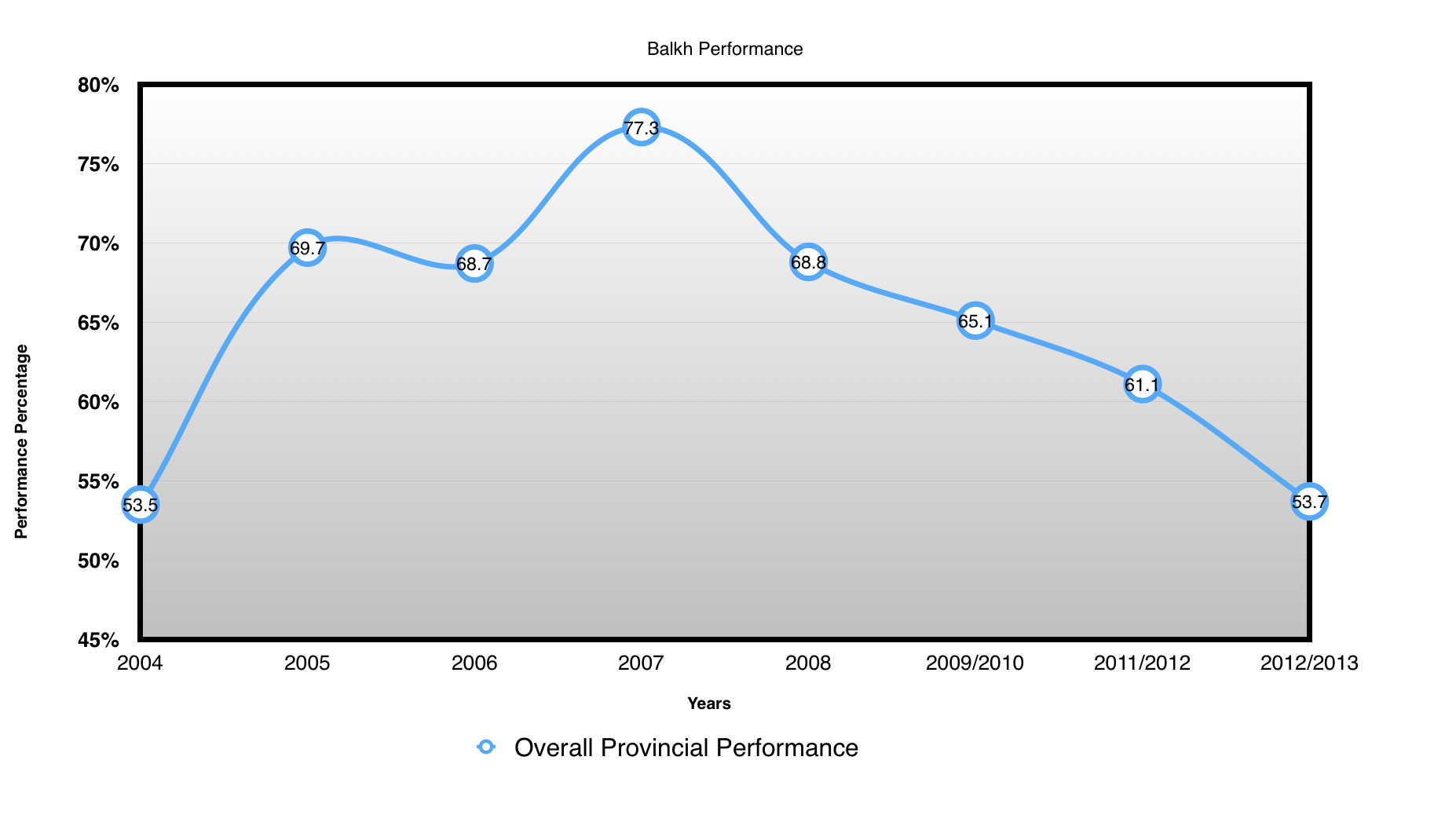


Chart 2

Balkh Province Composite Means Score Difference (Source BSC Reports)

The Balance scored card of 2012-2013 indicates that Balkh fell from 3rd to 24th place. This fall is more evident with the following indicators: Community Involvements and Decision-Making Index, Salary Payment Current, Revised Staff Received Training (over the past 12 months) 13. Revised Infrastructure Index, Client Counseling Index, Time Spent with Client, Revised HMIS Use Index, and Health Facility Management Functionality Index.

We notice that even if the donor and the provincial governance have not changed but there has been a declining curve in the performance of Balkh in the provision of health services. Key issues raised by the KII and FGDs in this province indicates that the handover from one Non-state provider to another (from BRAC to CHA) might have some impact.

### Herat

From 2003 to 2006 In Herat province health services have been contracted out to four different NSPs. Since it is rather large province, MOPH decided to divide it to four clusters and each cluster was contracted to a specific NSP. These NSPs are Coordination of Humanitarian Assistance (CHA) for C1, World Vision International (WVI) for C4. From 2006 to 2009 the services were contracted to CHA (C1) and MOVE (C2). The donor for the health services Bakhtar Development Network (BDN).

Except for few districts Herat province, in general, has been a calm place to provide health services. Politically the province has seen several governors from 2004 to 2013 and it is reported that all have had positive support towards health services. Geographically Herat is accessible in all seasons of the year and is not considered a mountainous place.

 The percentage of performance difference in lower benchmark has been improving 10.5 % between 2004 to 2013 (from 85% to 95.5%). The Balanced Scorecard of 2004 shows that the performance in this province has been YELLOW (patient satisfaction concentration index and HMIS Use Index were in RED) and in 2005 the province did show improvements in most of the benchmarks and was labeled YELLOW (but still Equipment Functionality Index and proper sharp disposal was in RED) with mean scores across indicators 1 through 22 of 53. In year 2006 it could keep its performance across all indicators with a little increase in means score as previous year (55.9). In 2007 comparing the last year again increased with means score of 69%. 2008 was the year did show decline comparing 2007 in most of the benchmarks across the indexes on domain of quality health services.


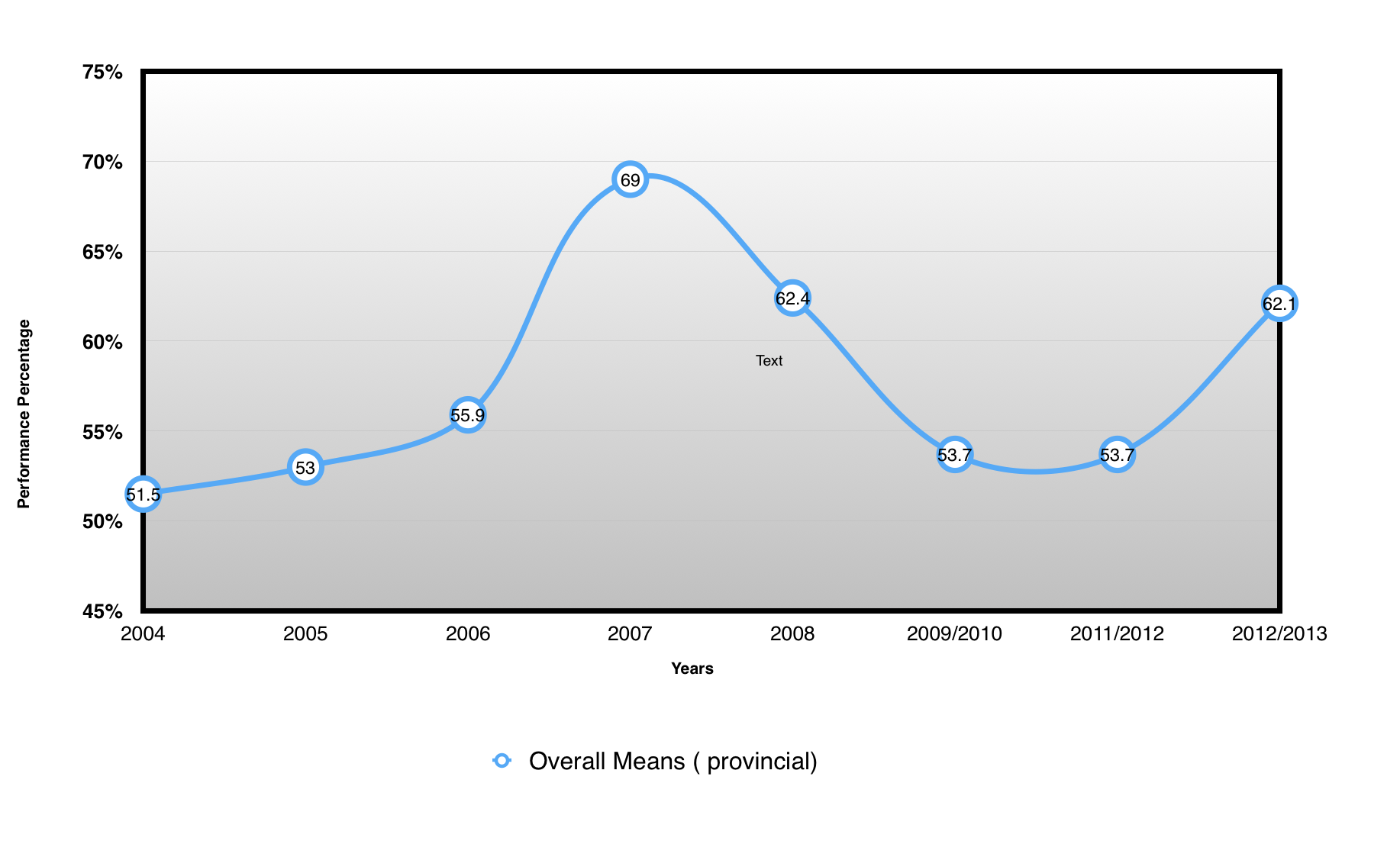


Chart 3

Herat Overall Means Score (Source BSC Reports)

In 2009 & 2010 it has still a higher mean score (71.3) in 2005 & 2011/2012 Herat fell behind (means score of 53 & 53.7 across most of the indicators). The patient satisfaction indicator, proper sharp disposal infrastructure, and functionality indexes were among the key indicators contributing to this fallback. In the following years, Herat fell back to the YELLOW and GREN areas. The Balance scored card of 2012-2013 indicates that Herat fell from 18th to 9th place. This rise is more evident with the following indicators: Community Involvements and Decision Making Index, Motivation, Drug and pharmaceutical, infrastructure, Revised HMIS Use Index, and Health Facility Management Functionality Index.

### Kandahar

In 2004 the Ministry of Public Health contracted out Basic Package of Health Services to an Afghan NSP, Afghanistan Health and Development Services (AHDS) which continued to deliver services till June 2015. The donor agency in all three cycles of contract out has been USAID.

The security has not been good in Kandahar during the past decade. Kandahar has had more security disturbances in border districts preventing smooth delivery of health services. Unfortunately, the trend has worsened over the past couple of years. Moreover, being a border province it is always prone to certain security issues. Geographically it is plain and hot. The arid and harsh climate of the province sometimes complicate the provision of health services during summer. Since traveling to Pakistan for health services is easy and better medical services and centers are available in Quetta patients to tend to choose to go to Pakistan for both minor and major health issues. Politically, the province has seen several governors from 2004 to 2013 and it is reported that all have had positive support towards health service.

 Due to heightened insecurity, the Third party could not conduct Balanced Scorecard data collection in Kandahar from 2005 to 2009. The percentage of a performance difference in lower benchmark has increased 6.8 % between 2004 to 2013 (from 75% to 81.8%). The mean score has reduced 10.4% (from 50.5to 60.9). Please see chart 5.


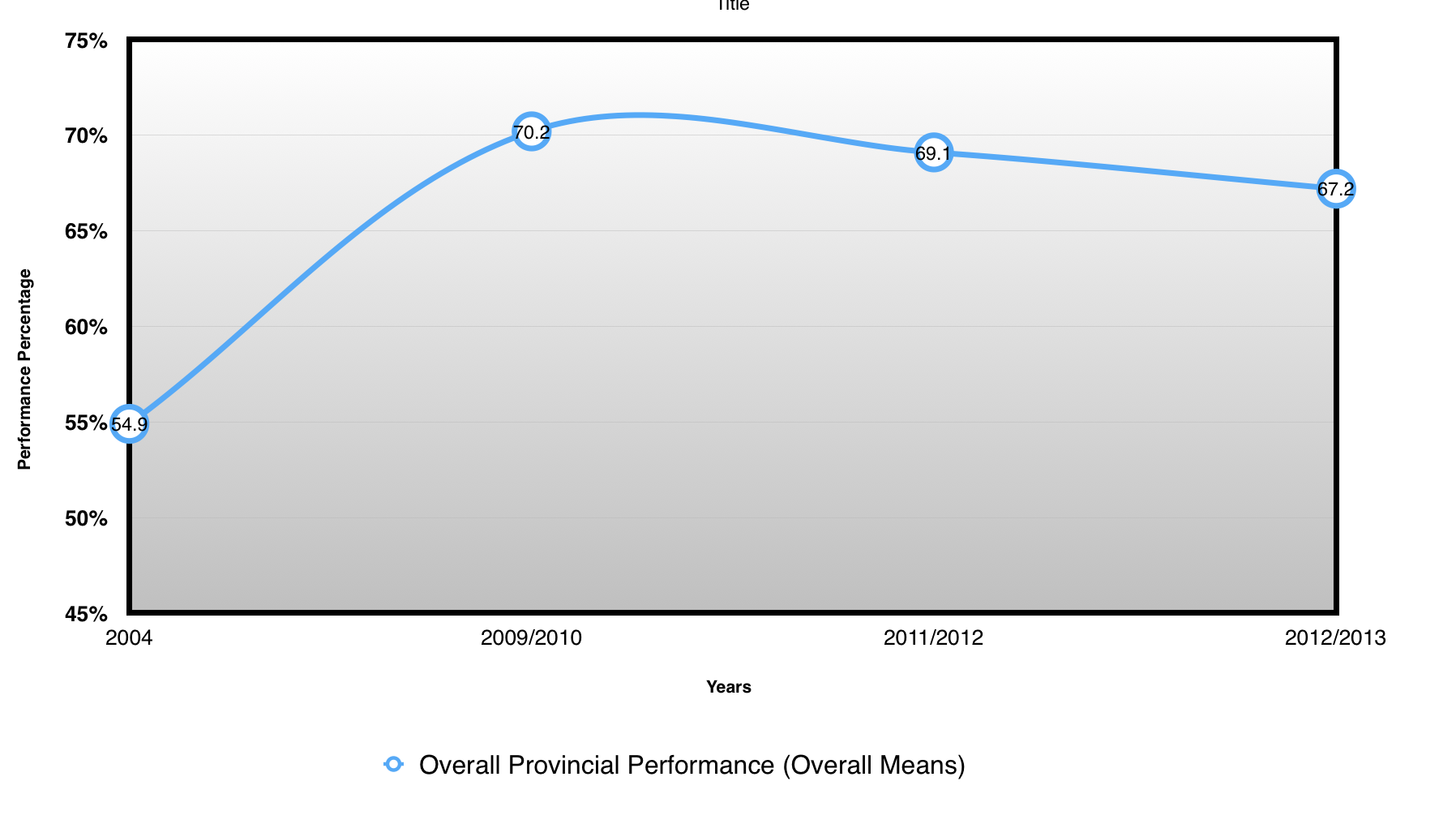


Chart 4

Kandahar BSC Means Score Differences (Source BSC Reports)

The Balanced Scorecard of 2004 shows that the performance in this province has been great for most of its indexes in both upper and lower levels (BSC 2004). Most of the indexes were in the YELLOW in 2004. From 2009 to 2013 all domains and indexes have shown great results mostly GREEN. In 2011/2012 Kandahar had no RED area at all with a mean score of 61.4.

Since 2013 we see a negative trend in some of the domains but again the domain of overall mission (patient satisfaction and outpatient visits) remained in RED. The Balance scored card of 2012-2013 indicates that Kandahar has slid down from 4th to 9^th^ place. This fall is more evident with the following indicators:  Health Worker Motivation Index, Provider Knowledge Score, Staff Received Training (in last year), Equipment Functionality Index, Pharmaceuticals and Vaccines Availability Index, Laboratory Functionality Index (CHCs only), Client Background and Physical Assessment Index, Client Counseling Index, Universal Precautions, HMIS Use Index, Financial Systems, Outpatient Visit Concentration Index, and Client Satisfaction Concentration Index

### Bamyan

From 2003 to 2006 In Bamyan province, health services have been contracted out to two different NSPs. Since it is rather a large province, MOPH decided to divide it into two clusters and each cluster was contracted to a specific NSP. These NSPs are Afghanistan Development and Reconstruction Agency(ADRA) for C2 and International Medical Corps (IMC) for C1. From 2006 to 2009 the services were contracted to AADA for (C1) and ADRA for (C2). The donor for the health services has always been USAID. In the third cycle (2009-2014) the entire province was contracted out to AADA. But in 2014 again it is divided into two clusters, cluster one was remained with AADA and cluster two was contracted with AKF.

The Security has been good. Except for a few districts Bamyan province in general has been a calm place to provide health services. Politically, the province has seen several governors from 2004 to 2013 and it is reported that all have positively supported health services. Geographically, Bamyan is a mountainous area located in central Afghanistan. Most of its districts is disconnected from t central Bamyan for several months during winter season making access a significant problem.

The percentage of the performance difference in lower benchmark has been declining 54.5 % between 2004 to 2013 (from 100% to 45.5%). The mean score has reduced 10.8% (from 60.6 to 49.8). Please see Chart 4.


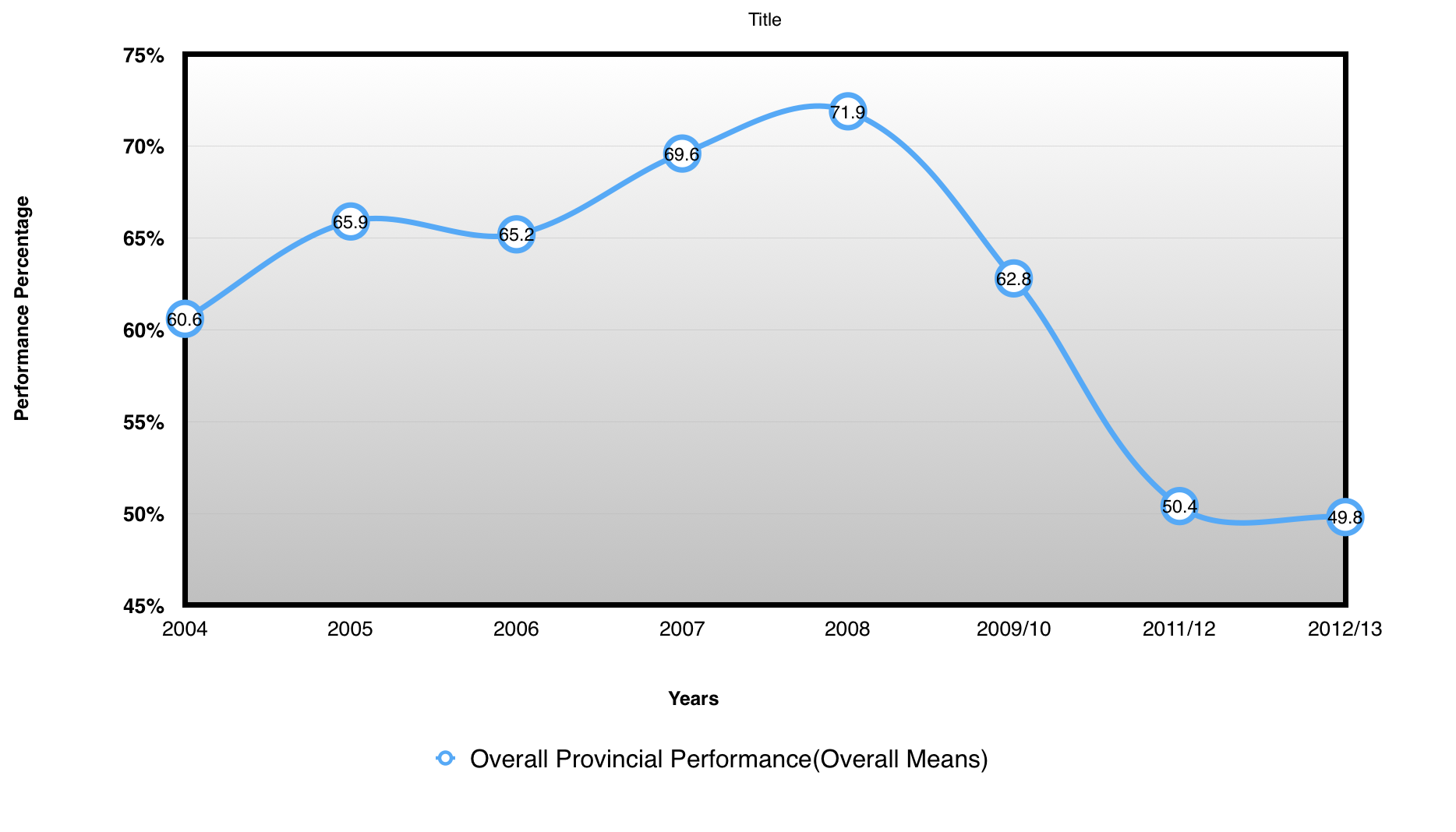


Chart 4

Bamyan BSC Means Score Differences (Source BSC Reports)

The Balanced Scorecard of 2004 shows that the performance in this province has been great for most of its indexes in both upper and lower levels (BSC 2004) but most of the indexes were in the YELLOW and in 2005 to 2008 the province did show improvements in most of the upper benchmarks and was labeled YELLOW and Green, but regarding lowest benchmark there is 5% fall.

Since 2009 to 2013 it is a falling trend among all component indexes. The Balance scored card of 2012-2013 indicates that Bamyan has slid down from (the 30^th^ to 31^st^ place). This fall is more evident with the following indicators:  Quality of care, Decision making, Satisfaction, Motivation, Salary payment current, Revised staff received training, Physical assessment, Counseling, proper sharp disposal and OPV (Oral Polio Virus) concentration index.

### Nangarhar

In 2003 the Ministry of Public Health contracted out Basic Package of Health Services to a Dutch NGO, Health Net International (Now called Health Net -TPO). Later during 2008 the EPHS was also covered under the contract out mechanism. EU during 2003-2014 directly paid the NSP and there was no intermediary. Since 2014 Nangarhar came under SEHAT project. Under SEHAT, BPHS was contracted to AADA and EPHS to Health Net TPO.

The security has not been good in Nangarhar all of the past year. Except for few years of serenity from 2004 to 2007 the province had security disturbances in the border districts preventing the smooth delivery of health services. Unfortunately, the trend is deteriorating over the past couple of years. Geographically it is a plain and tropical and does not pose many barriers in terms of implementation. However, being a border province it is always prone to certain security issues. On the other had medical travel is very common in Nangarhar. Since the travelling to Pakistan for health services is easy and better medical centers are available patients tend to choose to go to Pakistan for both minor and major health issues. Politically, the province has seen several governors from 2004 to 2013 and it is reported that all have positively supported health service.

The security has not been good in Nangarhar all of the past year. Except for few years of serenity from 2004 to 2007 the province had security disturbances in the border districts preventing the smooth delivery of health services. Unfortunately, the trend is deteriorating over the past couple of years. Geographically it is a plain and tropical and does not pose many barriers in terms of implementation. However, being a border province it is always prone to certain security issues. On the other had medical travel is very common in Nangarhar. Since the travelling to Pakistan for health services is easy and better medical treatment centers are available patients tend to choose to go to Pakistan for both minor and major health issues. Politically, the province has seen several governors from 2004 to 2013 and it is reported that all have positively supported t health service.

Nangarhar has been home to pilot of some key health interventions. The first Community Midwifery Education program was rolled out in Nangarhar. Moreover, incorporating Mental Health into Primary Healthcare first happened in Nangarhar.

The overall performance of Nangarhar has been good. The mean score has reduced 7.8% (from 54.9 to 67.2). Please see chart 3.


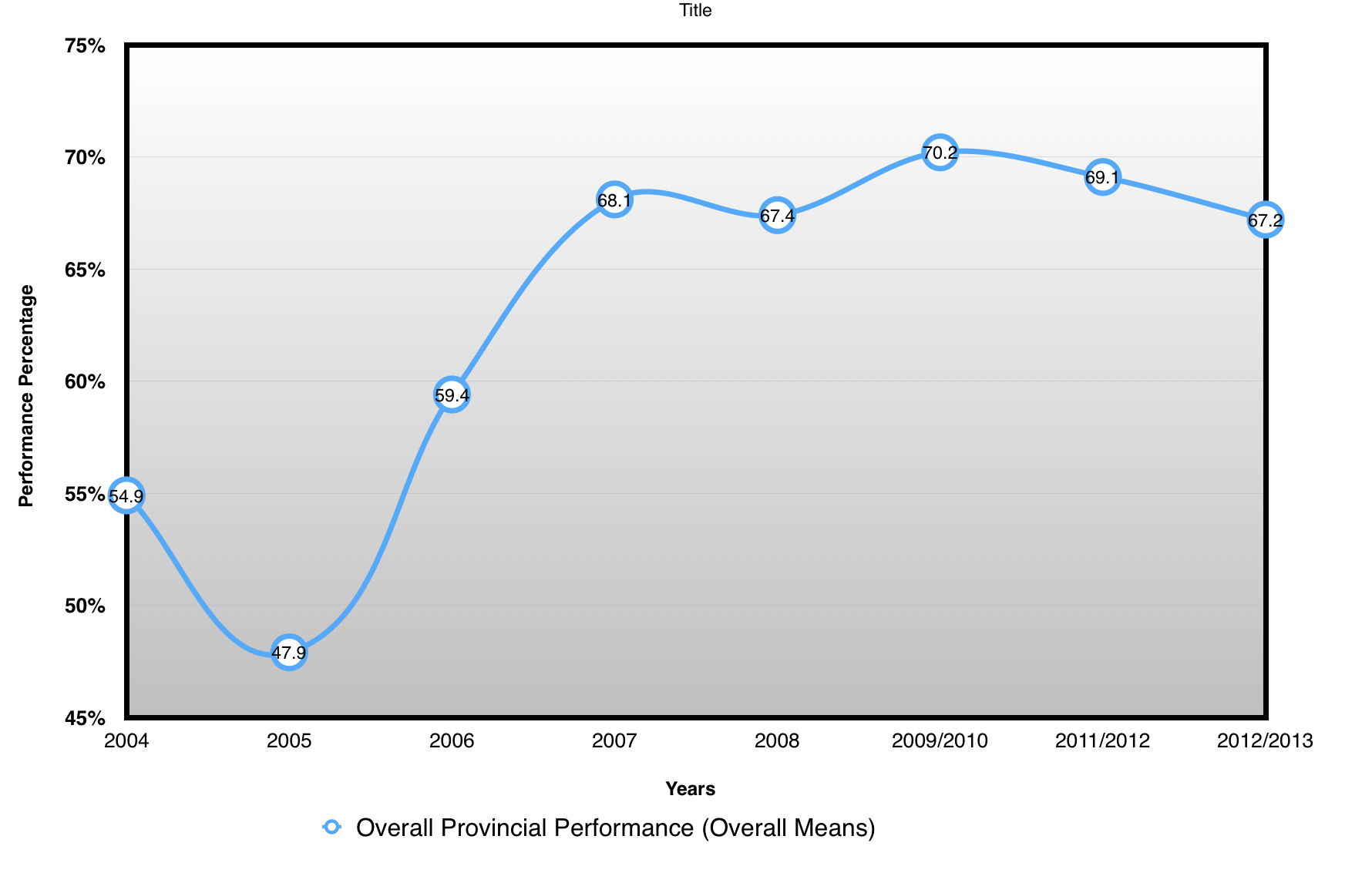


Chart 5

Nangarhar BSC Means Score Differences (Source BSC Reports)

The Balanced Scorecard of 2004 shows that the performance in this province has been great for most of its indexes (BSC 2004). Most of the indexes were in the GREEN from 2005 to 2008. All domains and indexes has shown great results till 2009 except for few exception (patient satisfaction and new patient visit concentration indexes).

Since 2009 to 2013 we see a positive trend in almost all of the domains but again the domain of overall mission (patient satisfaction and out-patient visits) remained in RED and Yellow. The Balanced Scorecard of 2012-2013 indicates that Nangarhar has slid down from 2^nd^ to 3^rd^ place. This fall is more evident with the following indicators:  Overall Client Satisfaction and Perceived Quality of Care Index, Staffing Index 8. Staff Received Training (in last year), Functional Infrastructure Index, HMIS Use Index and Financial Systems
